# Editorial: Discounting Models in Behavioral Health Economics and Quantitative Health Psychology

**DOI:** 10.3389/fpubh.2021.658808

**Published:** 2021-03-15

**Authors:** María José Muñoz Torrecillas, Salvador Cruz Rambaud, Taiki Takahashi

**Affiliations:** ^1^Department of Economics and Business, Universidad de Almería, Almería, Spain; ^2^Department of Behavioral Science, Center for Experimental Research in Social Sciences, Hokkaido University, Sapporo, Japan

**Keywords:** discounting models, behavioral economics, health economics, health psychology, public health

The purpose of this Research Topic is to encourage the research on time preferences (or discounting models) and their links with health-related behaviors or with policies to promote healthy behaviors, as shown by (1–4).

It has been suggested that behavioral economics is an important factor for improving the healthcare system [e.g., (5)], but relatively little progress has been made when compared to retirement policy. Some governments, like the UK and US, have made efforts to incorporate the behavioral economics approach into policy-making. The goal is to nudge citizens to increase organ donations or to encourage the payment of fines and taxes. There are examples of the successful applications of concepts from behavioral economics being implemented into public health policy to improve health attitudes and behaviors (e.g., the Behavioral Insights Team, the Penn Medicine Nudge Unit).

In the framework of time discounting, we can find time-inconsistent preferences (6), a departure from the rational choice model that implies a change in preferences. We see people failing in their attempts to quit smoking or to follow a diet despite having previously intended to do so. This is due to the fact that individuals, on many occasions, value what is available immediately more rather than a benefit that might come as a result in a distant future. Consequently, many individuals ignore the benefits of small behaviors with incremental effects that can avoid long-term adverse consequences. An example of a possible behavioral economic application in this situation would be to offer pre-commitment devices (4) that would allow people to reinforce self-control restricting the choices of their future selves, and increasing the probability of adhering to the healthy behavior.

This Research Topic consists of four articles, excluding this Editorial (three full length original research papers and one opinion paper). Their authors come from different areas of expertise such as Psychology, Economics, Behavioral Sciences, Health Education and Pediatric and Family Medicine and offer interesting applications of time discounting in the area of health economics (7).

Stegall et al. empirically study cross-cultural similarities and differences in social discounting for gain and losses. They compare four different quantitative social discounting models to gain and loss, and data from US, Japanese and German participants, concluding that the *q*-exponential function and the hyperbolic power function best fit median loss and gain data, respectively. They did not obtain significant absolute differences between cultures for gains or losses, and US participants showed a robust sign effect. Their results have important implications for health behaviors, such as addiction or obesity. For example, individuals that discount very steeply during a social discounting task may be at greater risk of drug use and/or eating disorders. Therefore, it is important to accurately quantify participants' social discounting behavior in order to use it as a prospective measure for health behavior risk, and/or a dependent variable for treatment.

Howatt et al. complete Muñoz-Torrecillas et al. (8) results and conclusions about the relationship between adherence to the Mediterranean Diet, considered a healthy dietary habit, and impulsivity in intertemporal choice. The results of Howatt et al. support their hypothesis that adherence to a healthy diet and greater self-control are connected. The importance of these results lie in the fact that dietary adherence and discount rates could potentially be used in conjunction to determine at-risk populations which should be targeted by health policies. More research is needed to expand on this work by analyzing and quantifying if people controlling their lifestyle and dietary habits may also be controlling impulsivity, and studying the efficacy of interventions to enhance the quality of life in both clinical and non-clinical populations.

Montoya Albrecht and Iyengar include an interesting economic perspective in the study of pediatric obesity that invites us to reflect on possible solutions to this problem, which is already an epidemic in developed countries. In the US, over thirty percent of American children are considered obese. As adults, many of these children will suffer from diseases such as diabetes, certain types of cancer, orthopedic conditions and depression, among others, resulting in a high level of government expenditure on public health and healthcare. The authors highlight the usefulness of behavioral economics and the understanding of discounting mechanisms in the development of treatments for many diseases, especially obesity. They encourage public interventions and propose increased taxation and bans on advertising of fast food, as in the tobacco industry, in order to eliminate problematic environmental triggers.

Berry et al. address the problem of poor air quality, also a major public health concern, relating the discount rate of air quality and visual exposure to natural vs. built environments. Their results showed that individuals exposed to natural scenes discounted improved air quality less, and this may be related to expanded space perception. These results show that viewing green spaces can cause people to be future-oriented in their approach to air quality decisions. Their findings may have implications for influencing environmentally relevant decision-making and improving individual and public health related outcomes such as air quality. More research is needed to establish if, for example, creating more natural spaces within cities could promote long-term oriented behavior and increase long-term environmental health.

Through these papers, we have explored some of the applications of the research on delay discounting to solve problems like poor air quality, smoking or obesity and the promotion of healthy lifestyle habits. These articles are the result of a growing interest in the applications of behavioral economics in the field of Health. We hope that this interest will continue to thrive and grow, given the benefits that this research can bring to the field of Policy and Health Economics and, in general, to individuals' health.

## Author Contributions

MJMT, SCR, and TT have jointly designed the research question, prepared the manuscript, and revised it. All authors contributed to the article and approved the submitted version.

## Conflict of Interest

The authors declare that the research was conducted in the absence of any commercial or financial relationships that could be construed as a potential conflict of interest.
